# Prevalence and Potential Risk Factors for Occupational Low Back Pain Among Male Military Pilots: A Study Based on Questionnaire and Physical Function Assessment

**DOI:** 10.3389/fpubh.2021.744601

**Published:** 2022-01-04

**Authors:** Yizhuo Yang, Shuai Liu, Mengyu Ling, Chaoqun Ye

**Affiliations:** ^1^Department of Rehabilitation Medicine, Air Force Medical Center, PLA, Beijing, China; ^2^Department of Rehabilitation Medicine and Physiotherapy, Anhui Medical University, Hefei, China

**Keywords:** low back pain, military pilots, functional test, transversus abdominis, risk factor

## Abstract

**Objectives:** Low back pain (LBP) has negative implications for the military's combat effectiveness. This study was conducted to determine the prevalence and risk factors of LBP among pilots through a questionnaire and physical function assessments.

**Methods:** Data on the demographic and occupational characteristics, health habits, physical activity, and musculoskeletal injuries of 217 male pilots (114 fighter, 48 helicopter, and 55 transport pilots) were collected using a self-reported questionnaire and physical function assessments.

**Results:** LBP prevalence was 37.8% in the total cohort and 36.0, 45.8, and 34.5% among fighter, helicopter, and transport pilots, respectively. Multivariate regression analysis revealed that the risk factors significantly associated with LBP were neck pain [odds ratio (OR): 3.559, 95% confidence interval (CI): 1.827–6.934], transversus abdominis activation (OR: 0.346, 95% CI: 0.172–0.698), and hip external rotator strength (OR: 0.001, 95% CI: 0.000–0.563) in the total cohort; neck pain (OR: 3.586, 95% CI: 1.365–9.418), transversus abdominis activation (OR: 0.268, 95% CI: 0.094–0.765), hip external rotator strength (OR: 0.000, 95% CI: 0.000–0.949), and weekly flying hours (OR: 3.889, 95% CI: 1.490–10.149) in fighter pilots; irregular strength training (OR: 0.036, 95% CI: 0.003–0.507) and hip external rotator strength (OR: 0.000, 95% CI: 0.000–0.042) in helicopter pilots; and neck pain (OR: 6.417, 95% CI: 1.424–28.909) in transport pilots.

**Conclusions:** High volume flight schedules and weak core muscle functions have significant negative effects on pilots' back health. LBP is commonly associated with high weekly flying hours, worsening neck pain, transversus abdominis insufficient activation, and reduced hip extensor/rotator strength. Risk factors vary among pilots of different aircraft. Thus, specific core muscle training would be especially important for military pilots.

## Introduction

The incidence of low back pain (LBP) among flight personnel has increased significantly due to the continuously increasing intensity of flight training (1–5), and one out of three pilots has reported LBP (6). LBP among pilots imposes significant challenges to the strength of the military. Spinal-related disorders such as LBP have become one of the highest causes of grounding for service pilots in China (7). In addition, LBP may affect pilots' attention and concentration (8), motor control (9), postural stability (9), and ultimately operational safety. This non-combat injury has become a major cause of troop attrition in modern warfare, so effective strategies must be found to reduce the high incidence of LBP in pilots.

Analyzing the relationship between pilots and LBP is complicated, and different aircraft vary greatly. Different types of aircraft have shown different prevalence of LBP (1) because of different +Gz forces (flying loads), whole-body vibration, and other properties. Specifically, fighter pilots have a high +Gz exposure (6, 10) and helicopter pilots are more affected by vibration during flight (11–14). Therefore, it is necessary to discuss the LBP problem in different aircraft separately. Some occupational factors, such as flight experience and flying hours (11, 15) and the fact that the duration of occupational exposure of pilots varies (1), may be other reasons for the occurrence of LBP. Though, compared with utility pilots, military pilots demand better physical function such as stronger core muscle to tolerate high load or prolonged confined sitting (1), but few studies in the literature have discussed these issues. Furthermore, previous studies investigating LBP within the last 12 months have reported different rates of LBP in the same type of aircraft, as recent and more severe pain may be remembered more clearly than earlier episodes of pain (16). Therefore, evaluating the incidence of LBP within the last 3 months may be more reliable.

Previous studies (16–18) have found that patients with LBP differ from healthy people in physical functional abilities such as hip mobility (19), low back muscle strength and endurance (16, 20, 21), and transversus abdominis activation (22, 23). Lifestyle factors, such as physical activities and other health habits, were also associated with occurrence of LBP (24). These predictive factors may be similar, but the impact of pilot-specific occupational characteristics should receive more attention. The physical functional abilities' assessments can guide the targeted training to prevent LBP. At present, the targeted training mainly focuses on the exercise of abdominis and back muscle strength, but ignores the core muscle functional training, such as transversus abdominis (TrA) activation. These related issues have not been mentioned in the recently published studies of the pilot's LBP, and therefore only through the analyses of personal and occupational information of the pilots and the assessment of physical function can the problematic parts of the current training be identified and improved.

This study aimed to identify the factors related to the development of occupational LBP in military pilots. It was hypothesized that the demographic and occupational characteristics, along with physical functions, were related to the incidence of LBP among pilots.

## Methods

### Study Design

This cross-sectional study was designed to investigate the prevalence of LBP among military pilots. The participants were recruited at the Airforce Medical Center and the data collection was started from July 2019 to January 2021. Before initiating this experiment, the study protocol was approved by the ethics committee of the Air Force Medical Center of People's Liberation Army of China (PLA) (Process No. 2020-150-PJ01). All participants were asked to complete the consent form in which they were informed about study aim and experimental procedures.

### Participants

Only male participants were recruited from the four Air Force military units and they were certified pilots aged between 20 and 55 years old. Participants who belong to any of the following criteria were excluded: (1) a current or past history of known spinal trauma, signs of neurological deficit, osteoarthritis, rheumatoid arthritis, or instrumental lesions, (2) suspended from flying for more than four consecutive weeks in the last 6 months (i.e., holiday, study, etc.). In total, 249 male participants who engaged in flying fighter, helicopter, or transport aircraft volunteered to participate in this study, but only 217 of them carried out the physical function assessment. The dropout data were: (1) 28 pilots were temporarily dispatched to other places; (2) two pilots withdrew because of injury during physical fitness test; and (3) two pilots had intolerable back pain caused by prolonged car driving.

### Questionnaire

Demographic information including age, height and weight (used to calculate body mass index (BMI) were collected. Participants were also asked to report additional data on occupational characteristics: (1) total flying hours (divided into four groups: <1000h, 1000–2000h, 2000–3000h and ≥3000h) and weekly flying hours (6 h was used a cut-off point: ≥6 h per week refers to high intensity while <6 h was defined as low intensity) in the past 6 months; (2) health habits (alcohol and smoking); (3) weekly strength training and core muscle training of >3 times; and (4) musculoskeletal injuries (as measured by the Nordic musculoskeletal questionnaire) (25). To minimize recall bias (16), pain experience in the last 3 months was measured, which was different from a previous study using the 12-month reports. LBP in this study was defined as pain symptoms that persisted for within the last 3 months, and the pain or discomfort affected daily flight schedules and required treatment (26).

### Physical Function Assessments

Physical function assessments contain the range of motion (ROM) of hip medial/lateral rotation, trunk muscle strength (isometric strength of trunk flexor and extensor) (27, 28), trunk muscle endurance [flexor endurance test, Sorensen test, and side-bridge test (21)], isometric strengths of hip muscles [extensors, abductors, and internal/external rotators (29)], and TrA muscle activation and balance test. Detailed procedure of each outcome was presented below.

The ROM of hip was measured using the clinical inclinometer as described by Eoghan et al. (30), which showed good test-retest reliability [intraclass correlation coefficient (ICC), 0.86]. Participants were asked to maintain the prone position with the measured hip placed in 0° of abduction and knee flexed to 90°. The inclinometer was placed in the distal tibia and adjusted to 0°; then the hip was allowed to do internal or external rotation until the contralateral pelvis lifted to stop. Repeat three times in each direction. The ROM was measured in the dominate leg, defined as the preferred leg for kicking a football in the lab, and the maximum value was reported.

The maximum isometric strengths of trunk and hip muscles were measured using a calibrated digital hand-held dynamometer (MicroFET 2, Hoggan Health Industries, USA) as described previously (27–29), which showed good test-retest reliability on measuring trunk and hip muscle strength (ICC, 0.86–0.97) (27, 29). It was held for 5 s in each test, and the average values of three replicates were used for data analysis. The maximum isometric strengths of the trunk included two tests: participants sat on a backrest chair with arms across the chest and feet off the ground; then they were fixed the body on the backrest by a strap and the hand-held dynamometer on their trunk by another strap; by the dynamometer was placed on the sternum stem/the height of the level with the fourth or fifth thoracic vertebra to allow the participant to resist bending forward/backward. The maximum isometric strengths of hip included four tests: the extensor required participants to maintain the prone position with hip extension 0° and knee flexion 90°, and the dynamometer was placed on the midline of the posterior thigh to allow the participant to resist extension; the abductor required participants maintain the supine position with hip extension 0°, and the dynamometer was placed on the lateral supra patella to allow the participant to resist abduction; the rotator required participants maintain the prone position with hip extension 0° and knee flexion 90°, and the dynamometer was placed on the lateral malleolus/medial malleolus to allow the participant to resist internal rotation/external rotation. The same tester (SL) performed all measurements to ensure consistency, and muscle strength testing order was randomized to minimize bias.

Trunk muscle endurance was measured using three tests (21). Firstly, the Flexor endurance test was used in which participants were asked to sit with the trunk risen to 60° from the bed with arms across the chest and the knees and hips flexed to 90°; the participant is asked to hold this position for as long as possible; the test ends when the body leans backwards at an angle of <60°. Secondly, the Sorensen test required participants to lie prone with the lower body fixed to the bed and upper body extended over the edge of the bed with the anterior superior iliac spine parallel to the edge of the bed; a chair was placed in front of the bed, and the arms were supported on the chair; when the test started, the participant lifted his arms away from the chair and crossed the chest, keeping the upper body on the horizon; if the upper body had a downward trend, it was allowed to remind once and return to the horizontal position, and the test would end at another descent. Thirdly, the Side-bridge test required participants to lie on their sides by left feet and left elbow supported with lifting the hips off the bed to maintain a straight line; if the pelvis had a downward trend, it was allowed to remind once and return to the straight position, and the test would end at another descent. During all tests, participants were reminded to maintain their position as long as possible.

TrA muscle activation (PRONE test) was assessed using a pressure biofeedback unit (Stabilizer Pressure Biofeedback Unit, Chattanooga Group Inc., USA) as described previously (31, 32), which showed good test-retest reliability (ICC =0.81) (31). This test was designed measure the ability of performing abdominal hollowing by holding the contraction for 10 s within 60–66 mmHg (70 mmHg began), and the score was expressed as contraction seconds (max value was 10). When the participant had compensatory movements such as rectus abdominis curling, pelvic forward tilt, hip flexion, or inability to breathe abnormally, or the pressure drops more than 10 mmHg or <4 mmHg during the 10s, the test ended. Previous studies have stated that the results of the PRONE test >2 s indicated the transverse abdominis can be activated (32), so participants were sub-grouped by the results into TrA activation group (≥3 s) and TrA inactivation group (<3 s). Unlike the other tests, each participant could have three to five attempts before the PRONE test.

The balance test was defined as standing on one foot without shoes with eyes closed, and the participant got a 0 point if he failed to remain balanced for <30 s, otherwise one point. When the participant was unable to maintain balance, such as jumping, raising the leg to the ground, or maintaining with external force, the test ended.

All tests were performed by the same test group (YY, ML, and SL) and the same tester performed the same measurements to ensure consistency (YY for ROM test, ML for PRONE test, and SL for muscle strength tests). The intra-tester reliability of measures was carried out before data collection, and intra-tester reliability was good with associations of intraclass correlation coefficient ranging from 0.80 to 0.92. No standard test protocol exists for the physical function assessments of pilots. It took about 90–120 min to complete the above tests in the same day. The order of all the test items was the balance test, the ROM tests of hip, the TrA muscle activation test, the maximum isometric strength tests of the trunk and hip muscles, and trunk muscle endurance tests. The maximum isometric strength tests and the trunk muscle endurance tests were intervals by no <20 min. Besides, these maximum isometric strength tests in each direction were intervals by no <60 s, and the three trunk endurance tests by no <5 min.

### Statistical Analysis

The data obtained through the questionnaire and LBP assessment were presented as mean with standard deviation (SD) and as absolute values with percentages. Data normality was tested using the Kolmogorov–Smirnov test. Normally distributed data were evaluated using One way ANOVA, and a chi-square test was used for non-normally distributed data. Then we conducted a Bonferroni's *post-hoc* analysis to identify changes among aircraft. A binary logistic regression of variables with *p* < 0.20 was used to calculate the multivariate odds ratio (OR) and 95% confidence interval (CI) according to the results of univariate regression analysis. A stepwise backward elimination procedure was used to determine the optimum regression equation. For a term to be retained, the term should significantly contribute to the prediction of y (p <0.05). Multivariate regression was adjusted for age and total flying time. Using the logistic regression model, the possibility of incidence of LBP (y) based on the independent variable (x) was calculated depending on questionnaire data and physical function data where A are regression coefficients and B are dummy variables, and the full regression model was:


(1) $$\begin{array}{r} \text{y}=\text{Ax}+\text{B} \end{array}$$

Collinearity was checked all the related independent variable which a correlation coefficient <0.50. All analyses were performed using SPSS version 17.0 (IBM Corporation, USA). A *p*-value of < 0.05 was considered to be statistically significant.

## Results

### Participant Characteristics

A total of 217 male pilots (age: 36.7 ± 7.6 years; BMI: 24.12 ± 2.13 kg/m^2^), including 114 fighter pilots, 48 helicopter pilots, and 55 transport pilots, volunteered to participate in this study. The demographic data and other characteristics were displayed in Table 1. No group differences were observed in the demographic data, occupational features, and the pain location of spine. Total flying hours was 2197.4 on average, and pilots of different aircraft were distributed differently in hours groups. Fighter pilots (36.0%) were mainly distributed in 1,000–2,000 h, but helicopters (62.6%) and transports (43.7%) above 2000 h. Weekly flying hours in the past 6 months was 7.3 and 60.4% of helicopter pilots over 6 h. Physical functions were presented in Table 2. Significant group differences were detected in fle/ext strength (*p* = 0.040), Sorensen test (*p* < 0.001), side bridge (*p* = 0.012), hip extensor strength (*p* < 0.001), hip abductor strength (*p* = 0.035) and hip external rotator strength (*p* = 0.024). Results from further comparison indicated that the fighter pilots showed significantly higher fle/ext strength (*p* = 0.035), side-bridge score (*p* < 0.001), hip extensor strength (*p* < 0.001) and hip external rotator strength (*p* = 0.042) than those of the helicopter pilots. The fighter pilots had a significantly higher Sorensen test score (*p* < 0.001), hip extensor strength (*p* < 0.001) and hip abductor strength (*p* = 0.039) than those of the transport pilots.

**Table 1:** Demographic and other characteristics of the study population.

			**Aircraft category**			
		**Total**	**Fighter pilots**	**Helicopter pilots**	**Transport pilots**	***p*-value**
		**(*N* = 217)**	**(*n* = 114)**	**(*n* = 48)**	**(*n* = 55)**	
Demographic data	Age (year)	36.7 (7.6)	36.6 (6.0)	37.2 (7.9)	36.6 (10.0)	0.901
	Height (cm)	174.5 (7.8)	174.3 (10.0)	175.1 (4.2)	174.4 (4.2)	0.829
	Weight (kg)	73.4 (6.9)	72.6 (5.6)	74.6 (8.0)	74.0 (8.0)	0.201
	BMI (kg/m^2^)	24.2 (2.1)	24.3 (1.9)	24.0 (2.1)	24.2 (2.5)	0.826
	<24 (*n*)	99 (45.6)	49 (43.0)	21 (43.8)	29 (52.7)	
	≥24 (*n*)	118 (54.4)	65 (57.0)	27 (56.3)	26 (47.3)	
	Smoking (*n*)	112 (51.6)	62 (54.4)	29 (60.4)	21 (38.2)	0.055
	Alcohol (*n*)	69 (31.8)	33 (28.9)	18 (37.5)	18 (32.7)	0.557
Occupational data	Total flying hours (h)	2197.4 (1563.7)	2019.7 (1102.2)	2324.0 (1722.9)	2455.5 (2122.7)	0.194
	<1000 (*n*)	47 (21.7)	17 (14.9)	11 (22.9)	19 (34.5)	
	≥1000, <2000 (*n*)	60 (27.6)	41 (36.0)	7 (14.6)	12 (21.8)	
	≥2000, <3000 (*n*)	49 (22.6)	30 (26.3)	15 (31.3)	4 (7.3)	
	≥3000 (*n*)	61 (28.1)	26 (22.8)	15 (31.3)	20 (36.4)	
	Week flying hour (h)	7.3 (7.1)	8.16 (7.9)	6.2 (6.4)	6.3 (5.4)	0.137
	Low-weekly-hour (*n*)	126 (58.1)	71 (62.3)	19 (39.6)	36 (65.5)	
	High-weekly-hour (*n*)	91 (41.9)	43 (37.7)	29 (60.4)	19 (34.5)	
Physical activity	Regular strength training (*n*)	156 (71.9)	84 (73.7)	38 (79.2)	34 (61.8)	0.122
	Regular core muscle training (*n*)	85 (39.2)	49 (43.0)	21 (43.8)	15 (27.3)	0.112
Pain location	Neck (*n*)	54 (24.9)	34 (29.8)	10 (20.8)	10 (18.2)	0.199
	Upper back (*n*)	20 (9.2)	12 (10.5)	4 (8.3)	4 (7.3)	0.768
	Lower back (*n*)	82 (37.8)	41 (36.0)	22 (45.8)	19 (34.5)	0.421

*Presented as mean (standard deviation) or as absolute values (percentages)*.

**Table 2:** Physical functions in the study population.

		**Total**	**Aircraft category**			
			**Fighter pilots**	**Helicopter pilots**	**Transport pilots**	***p*-value**
		**(*N* = 217)**	**(*n* = 114)**	**(*n* = 48)**	**(*n* = 55)**	
Hip ROM (°)	Medial rotation	26.6 (7.4)	25.3 (8.2)	26.7 (5.8)	29.2 (6.3)	0.657
	Lateral rotation	40.0 (11.0)	36.3 (11.8)	43.7 (9.2)	44.3 (7.5)	0.059
Fle/ext strength (%BW)		0.98 (0.37)	1.03 (0.34)	0.87 (0.36)^*^	0.99 (0.41)	0.040
Endurance (s)	Flexor endurance test	104.2 (50.3)	108.4 (48.4)	102.1 (53.3)	97.4 (51.6)	0.391
	Sorensen test	88.0 (31.7)	95.1 (28.4)	85.8 (38.8)	75.1 (27.5)^#^	<0.001
	Side bridge	65.5 (24.5)	70.1 (24.5)	58.9 (21.5)^*^	61.7 (25.4)	0.012
PRONE test (s)		2.6 (4.0)	2.9 (4.2)	2.3 (3.8)	2.4 (3.7)	0.641
	TrA activation (*n*)	152 (70.0)	76 (66.7)	35 (72.9)	41 (74.5)	
	TrA inactivation (*n*)	65 (30.0)	38 (33.3)	13 (27.1)	14 (25.5)	
Hip strength (%BW)	Extensor	0.35 (0.10)	0.38 (0.09)	0.31 (0.08)^*^	0.33(0.10)^#^	<0.001
	Abductor	0.20 (0.05)	0.21 (0.05)	0.19 (0.06)	0.19 (0.04)	0.035
	Internal rotator	0.16 (0.05)	0.16 (0.05)	0.14 (0.04)	0.16 (0.05)	0.100
	External rotator	0.17 (0.04)	0.18 (0.05)	0.16 (0.04)^*^	0.16 (0.04)^#^	0.024
Balance test (*n*)		102 (47.0)	49 (43.0)	23 (47.9)	30 (54.5)	0.366

*Presented as mean (standard deviation) or as absolute values (percentages)*.

* *Indicated significant difference between flighter and helicopter pilots*.

# *Indicated significant differences between flighter and Transport pilots*.

### Factors Associated With LBP

The risk factors for LBP were shown in Table 3.

**Table 3:** Risk factors for low back pain in the study population.

			**Univariate**			**Multivariate** ^**a**^	
**Pilot category**	**Variables**	**β**	**OR (95% CI)**	***p*-value**	**β**	**OR (95% CI)**	***p*-value**
Total cohort	Irregular strength training	0.508	0.602 (0.319–1.137)	0.118			
	Neck pain	1.296	3.656 (1.925–6.943)	<0.001	1.269	3.559 (1.827–6.934)	<0.001
	Upper back pain	1.237	3.445 (1.313–9.037)	0.012			
	Side bridge endurance	−0.010	0.990 (0.978–1.001)	0.080			
	TrA activation	−0.996	0.381 (0.197–0.736)	0.004	−1.060	0.346 (0.172–0.698)	0.003
	Hip strength						
	Extensors	−2.402	0.090 (0.005–1.747)	0.112			
	Internal rotators	−7.343	0.001 (0.000–0.251)	0.016			
	External rotators	−7.477	0.001 (0.000–0.300)	0.019	−7.131	0.001 (0.000–0.563)	0.033
Fighter pilots	Age	0.247	1.050 (0.983–1.121)	0.149			
	BMI ≥ 24	0.575	1.776 (0.804–3.922)	0.155			
	Total flying hours	0.775	1.000 (1.000–1.001)	0.174			
	High–weekly-hour	1.053	2.867 (1.293–6.355)	0.009	1.358	3.889 (1.490–10.149)	0.006
	Irregular core muscle training	0.523	0.593 (0.274–1.284)	0.185			
	Neck pain	1.390	4.014 (1.724–9.346)	0.001	1.277	3.586 (1.365–9.418)	0.010
	Upper back pain	1.431	4.182 (1.175–14.889)	0.027			
	TrA activation	−1.057	0.347 (0.141–0.857)	0.022	−1.316	0.268 (0.094–0.765)	0.014
	Hip strength						
	Extensors	−5.149	0.006 (0.000–0.613)	0.030			
	Internal rotators	−11.038	0.000 (0.000–0.081)	0.011			
	External rotators	−12.648	0.008 (0.000–0.038)	0.008	−11.032	0.000 (0.000–0.949)	0.049
Helicopter pilots	Height	−0.107	0.898 (0.779–1.037)	0.143			
	BMI ≥ 24	0.916	2.500 (0.766–8.160)	0.129			
	Irregular strength training	−2.409	0.090 (0.010–0.782)	0.029	−3.317	0.036 (0.003–0.507)	0.014
	Flexor endurance test	−0.011	0.989 (0.976–1.002)	0.098			
	Hip internal rotator strength	12.110	0.000 (0.000–0.618)	0.125	20.386	0.000 (0.000–0.042)	0.020
Transport pilots	High-weekly-hour	−0.985	0.373 (0.103–1.352)	0.133			
	Neck pain	1.859	6.417 (1.424–28.909)	0.016	1.859	6.417 (1.424–28.909)	0.016
	TrA activation	−0.853	0.426 (0.103–1.767)	0.240			

a *Adjusted for age and total flying time*.

*Adjusted R^2^ of the total cohort = 0.412; adjusted R^2^ of fighter pilots = 0.319; adjusted R^2^ of helicopter pilots = 0.183; adjusted R^2^ of transport pilots = 0.194*.

The unadjusted and adjusted analyses with linear regression models for the LBP were reported in Table 3. The multivariate regression analysis revealed that some factors significantly predicted LBP. The full regression model of (1) the total cohort, (2) the flight pilots, (3) the helicopter pilots, and (4) the transport pilots were (y_1_: the total cohort, y_2_: the flight pilots, y_3_: the helicopter pilots, y_4_: the transport pilots; x_1_: neck pain, x_2_: TrA activation, x_3_: hip strength of external rotator, x_4_: hip strength of internal rotator, x_5_: high-weekly-hour, x_6_: Strength training irregularly):


(2) $$\begin{array}{r} {\text{y}}_{1}=1.269{\text{x}}_{1}-1.060{\text{x}}_{2}-7.131{\text{x}}_{3}+1.692 \end{array}$$

(3) $$\begin{array}{r} {\text{y}}_{2}=1.277{\text{x}}_{1}+1.358{\text{x}}_{5}-1.316{\text{x}}_{2}-11.032{\text{x}}_{3}+3.168 \end{array}$$

(4) $$\begin{array}{r} {\text{y}}_{3}=20.386{\text{x}}_{4}-3.317{\text{x}}_{6}+6.586 \end{array}$$

(5) $$\begin{array}{r} {\text{y}}_{4}=1.859{\text{x}}_{1}-1.012 \end{array}$$

## Discussion

Non-combat injuries are the leading cause of pilot attrition and military discharge. Back pain, especially LBP (approximately 75% of cases), is the most common complaint in military personnel and appears to increase during training and combat deployments (33). The present study tried to identify the risk factors of occupational LBP among pilots by analyzing demographic, occupational, and muscle function data. The main finding was that LBP of pilots was positively related to neck pain history and weekly flying hours, but negatively related to TrA muscle activation and hip strength. Furthermore, these factors varied among pilots of different aircraft types. These findings may provide new insights into the pilot training.

### Epidemiology of LBP in Aircraft Pilots

In this study, the prevalence of LBP was 37.8% in the total cohort and 36.0%, 45.8%, and 34.5% in fighter, helicopter, and transport pilots, respectively. The rate of back pain is high among military pilots, from 32 to 89% (1, 2, 4, 5, 11, 15, 34). Besides, vibration frequencies and +Gz gravity loads vary among aircraft types and are the influential factors of LBP (6, 14). The prevalence of LBP is remarkably higher among utility and attack helicopter pilots (89.38% and 74.55%, respectively) than among fighter and transport pilots (64.02% and 47.47%, respectively) (1). Lis et al. (35) found that helicopter pilots had the highest incidence of LBP among all occupations. Compared with previous studies, the LBP reporting rate in this study is relatively low, and the results showed no significant differences in different aircraft. The prevalence of LBP was lower in our cohort because the more stringent definition of LBP increased the credibility of the results to some extent. This finding is corroborated by another study in which the 3-month prevalence of LBP is slightly lower than the 12-month prevalence (11).

### Can Demographic Characteristics Predict LBP in Pilots?

As people age, inevitably increased BMI and its age-related functional decline can contribute to the higher risk of developing LBP (17), but the relationship between demographics and LBP in pilots is inconclusive. A recent study showed that age is a risk factor for LBP in pilots (4). By contrast, we found no correlation between these factors which are consistent with other reports (5, 11). The mean BMI of pilots was higher than 24, which might be due to high muscle mass. Moreover, contradictory to the findings of previous investigations (5, 13), there was no significant association between height and LBP in the present study.

Regular strength training is the foundation of good physical performance and core muscle strengthening training can decrease the risk of back pain (17). The findings showed a good awareness of strength training among pilots of which a large percentage of our cohort practice strength training regularly, and this percentage was higher than that of a 2012 survey (50%) (10). However, the number of participants that perform core muscle training was low. We also found that the concept of core muscle training was unclear to the pilots, which may explain why the results are contrary to the hypothesis. Most of them believed that core muscle strengthening engaged the rectus abdominis (sit-ups) and erector spinae (back extension). This misconception may lead to inadequate training. A paradoxical finding was that irregular strength training was a protective factor for LBP in helicopter pilots, which might be due to the fact that these pilots were more aware of the importance of exercise as evidenced by the fact that 75% performed strength training regularly regardless of the presence of LBP. Another reason might be that weak deep postural muscles lead to superficial muscles overactivation, so general strength training without kinetic control may be counterproductive to the protection of the spine (36).

Neck pain and upper back pain were independent risk factors for LBP in the total cohort and in fighter pilots; neck pain was a negative predictor of LBP in the multivariate regression analysis and was the greatest predictor of LBP in transport pilots. This finding indicates that neck pain, upper back pain, and LBP are interconnected (33). The spine should be viewed as a whole. The fatigue of the spine muscles during flight may change the sitting posture, forcing the back to bend more. To maintain trunk balance, the pilot may need to forward his neck further. This series of compensatory actions may make the pilot's spine more susceptible to pain. Therefore, we should be alert to the possibility of LBP in a patient with neck pain and vice versa.

### Is LBP Associated With Flying Experience?

In our study, the number of flying hours per week, but not total flying time, was associated with LBP. The total number of flying hours reflects the exposure time to a particular occupational environment. Exposure time is related to LBP (37). Long-term exposure associated with high-load pressure results in muscle contraction and potentially leads to musculoskeletal disorders, especially LBP and neck pain. Recent studies established that an increased prevalence of LBP was mainly associated with pilots' long flying time (1, 5, 33, 37). However, some studies reported inconsistent findings (6). Flying more than 6 h a week increased the risk of LBP in fighter pilots because of continuously high +Gz exposure during flight and insufficient recovery time. We found that the average total flying time of fighter pilots is the least of the three aircraft, but their average weekly flying time is indeed the longest. This finding also confirms the effect of long weekly working hours on pilots. Pilots flying the same type of aircraft may be exposed to different factors depending on the military mission and flight schedule; therefore, discussing total flying hours only is insufficient. The increased number of flying hours per week in our cohort can reflect the level of occupational exposure in the last 6 months and is therefore a better indicator of chronic musculoskeletal pain. Vibration is considered to be a characteristic of helicopters, but results from different studies were contradictory (1, 5, 11, 37). The whole-body vibration is transmitted to the entire body through the seat, resulting in a reduction in muscle fatigue resistance, then leading to LBP in helicopter pilots (38). In our study, the prevalence of LBP was higher in helicopter pilots than the other two types of aircraft; however, no relationship was found between exposure (total-flying-hours and week-flying-hours) and LBP. So, the exposure time to vibration may be as an independent indicator for helicopter pilots which is worth to be discussed in future studies.

### Does Improvement in Core Muscle Strength Reduce the Incidence of LBP in Pilots?

Research support for designing targeted training programs is lacking because literature data on the physical functions of military pilots are limited. The present study evaluated physical functions that might contribute to LBP in military pilots and found that pilots of different aircraft had different functional performance. We also found that TrA muscle function and hip rotator strength significantly impacted LBP. Thus, the results provide new ideas for the targeted physical training of military pilots.

Our study showed that the core muscle strength of pilots showed differences in different aircraft. The fle/ext strength test found that fighter pilots' abdominal muscle strength was significantly better than back muscle strength, and the side-bridge test results were also better than other models. These tests measured core stability and endurance (21, 22) which indicated that fighter pilots showed better core muscle function due to their anti-G acceleration requirements during flight (6, 10). The Sorensen test found that the endurance of back muscles of transport aircraft pilots was significantly weaker, and the performance in other tests were also weak. This may be due to the fact that work as transport aircraft pilot is less physically demanding than work as a fighter or helicopter pilot (1).

The TrA is an important respiratory muscle and deep postural muscle and plays an important role in maintaining core trunk stability (39). Respiratory muscle fatigue affects anti-G respiratory maneuver training; for this reason, core muscle strengthening is crucial (40). We found that TrA muscle activation was associated with LBP, and the risk of LBP was significantly reduced in the pilots who had a better TrA function. A study found no difference in the TrA thickness between the patients with recurrent LBP and healthy controls (22). However, Hagin et al. (23) reported that the respiratory exertion of individuals with LBP was higher when lifting heavy weights; this finding suggests that the relationship between TrA and LBP may be due to TrA dysfunction. Based on clinical findings, weakness of the deep postural muscles can lead to overcompensation of the superficial large muscles, which can further lead to impaired muscle motor control (22, 41, 42). Therefore, low-load motor control training is an integral part of the solution to chronic pain compared to endurance and strength training. A study by Salmon et al. (36) stated that training of the deep postural muscles was effective in improving neck pain in helicopter pilots. Similarly, a randomized controlled study found that the prevalence of LBP is lower among US Air Force pilots who performed specific core muscle exercises regularly vs. a control group that did not perform these exercises (43). The advantages of TrA were more pronounced in our cohort. The results indicate the importance of good TrA function for LBP prevention and suggest that the TrA improves tolerance to +Gz loads. Therefore, biofeedback training and strengthening exercise in core muscle training are essential to improve the TrA function of pilots for LBP prevention.

Previous studies focused on the influence of hip extensors and abductors on LBP (19). We found that the strength of hip extensors and internal/external rotators were associated with LBP in pilots and might be related to flying posture, as the pilots remain in a seated position and maintain their pelvis stable, which reduce the demand on hip abductors and increase the demand for push–pull movements by the upper limbs. Moreover, internal and external hip rotators are involved in the force transfer along myofascial chains (44). The hip rotators are part of the functional line and spiral line, which transfer the power of the lower body to the upper body and provide core stability (45). Therefore, this reason can also explain hip muscle weakness associated with LBP in the context of myofascial chains. Hip flexor strength was an independent risk factor for LBP and might be related to tolerance to +Gz acceleration. Therefore, physical training should focus on improving hip strength. Factors associated with LBP, including trunk flexor/extensor strength ratio (20), did not increase the risk of LBP in our participants probably because of occupational and physical function characteristics.

An effective approach to reduce the incidence of LBP is making ergonomic changes to the aircraft, however the usefulness of such changes is controversial. Therefore, LBP prevention should focus on physical training, which is critical in the short term. Targeted training improves core muscle strength, reduces the workload of chronically fatigued muscles such as the erector spinae and quadratus lumborum, and further reduces the risk of chronic LBP (17). However, no evidence-based guidelines or consensus for LBP prevention in Chinese pilots are currently available. Developing training programs to improve physical functions in military pilots and assessing the effects of these interventions are fundamental.

### Strengths and Limitations

The study population was representative because the participants came from different air force units in China and flew different aircraft types. Hence, the study population can provide a broad perspective on LBP for PLA Air Force researchers. Moreover, the physical function results demonstrated the presence of muscle weakness and biomechanical problems in this population. The entire body should function optimally to maximize performance. These findings can provide a basis for developing training programs to prevent or reduce LBP.

This study has limitations. First, our study had recruited over 200 pilots who fly different types of aircraft. The sample sizes for helicopters and transport aircraft are relatively small and should conduct large-sample study in the future. Second, the loss of follow-up might lead to bias. Third, this study was a part of a larger study and the use of a uniform questionnaire while the participants were surveyed may have resulted some targeted information being missed. Additionally, some details related to LBP were absent, such as active lifestyle habits and flexibility work, so the survey study should be enhanced in the next phase of the intervention study. We also admitted that the physical functional assessments related to LBP may be inadequate, and it would be improved in the future research. Finally, the cross-sectional nature of the study does not allow the determination of causality because evidence of the temporal relationship between the risk factors and outcomes is lacking. Therefore, long-term follow-up studies are necessary to assess the causes of LBP in pilots.

## Conclusions

Demographic indicators were not significantly related to LBP in military pilots. Several strategies can be adopted to reduce LBP in this population, such as establishing adequate flight schedules to improve rest and avoid fatigue and strengthening hip rotators and core muscles, in particularly, the transversus abdominis function. In addition, the interaction between neck pain and LBP should be the focus of future research, and a holistic view of spinal protection is needed.

## Data Availability Statement

The original contributions presented in the study are included in the article/supplementary material, further inquiries can be directed to the corresponding author.

## Ethics Statement

The studies involving human participants were reviewed and approved by the People's Liberation Army of China (PLA) Air Force Medical Center (Process No. 2020-150-PJ01). The patients/participants provided their written informed consent to participate in this study.

## Author Contributions

CY and YY wrote and revised the research proposal and statistical analysis plan. YY, ML, and SL collected, managed, and analyzed the data and drafted and revised the manuscript. CY is the guarantor and manager. All authors contributed to the article and approved the submitted version.

## Funding

Funding support was provided by Air Force LSD, China (2017-651).

## Conflict of Interest

The authors declare that the research was conducted in the absence of any commercial or financial relationships that could be construed as a potential conflict of interest.

## Publisher's Note

All claims expressed in this article are solely those of the authors and do not necessarily represent those of their affiliated organizations, or those of the publisher, the editors and the reviewers. Any product that may be evaluated in this article, or claim that may be made by its manufacturer, is not guaranteed or endorsed by the publisher.

## Abbreviations

LBP: low back pain
PLA: People's Liberation Army of China
BMI: body mass index
TrA: transversus abdominis
SD: standard deviation
OR: odds ratio
CI: confidence interval.

## Appendix

**Questionnaire (English Version)**

**Part one**

1. Sex: □male □female
2. Date: xx/xx/xxxx
3. Height: ___cm
4. Weight: ___kg
5. Smoking: □yes □no
6. Alcohol: □yes □no
7. Aircraft type: □fighter, ____; □helicopter, ____; □transport, ____; □other, _____
8. Total flying hours: ____h
9. In the past 6 months, week flying hour: ____h
10. Strength training habits: □yes □no (defined that training at least three times a week and each time not <30 min.)
11. Core muscle training habits: □yes □no (defined that training at least three times a week and each time not <30 min.)

**Part two**

Nordic Questionnaire
